# Elevation patterns and critical environmental drivers of the taxonomic, functional, and phylogenetic diversity of small mammals in a karst mountain area

**DOI:** 10.1002/ece3.6750

**Published:** 2020-09-09

**Authors:** Jian Sun, Zhixin Wen, Anderson Feijó, Jilong Cheng, Yanqun Wang, Song Li, Deyan Ge, Lin Xia, Qisen Yang

**Affiliations:** ^1^ Key Laboratory of Zoological Systematics and Evolution Institute of Zoology Chinese Academy of Sciences Beijing China; ^2^ University of Chinese Academy of Sciences Beijing China; ^3^ Kunming Institute of Zoology Chinese Academy of Sciences Kunming China

**Keywords:** biodiversity, climate factors, elevation gradient, human influence

## Abstract

Understanding how biodiversity components are related under different environmental factors is a fundamental challenge for ecology studies, yet there is little knowledge of this interplay among the biotas, especially small mammals, in karst mountain areas. Here, we examine the elevation patterns of the taxonomic diversity (TD), phylogenetic diversity (PD), and functional diversity (FD) of small mammals in a karst mountain area, the Wuling Mountains, Southwest China, and compare these patterns between taxa (Rodentia and Eulipotyphla) and scales (broad‐ and narrow‐range species). We also disentangle the impacts of the human influence index, net primary productivity (NPP), normalized difference vegetation index (NDVI), annual precipitation (AP), and annual mean temperature (AMT) on these three facets of biodiversity by using structural equation modeling. We recorded a total of 39 small mammal species, including 26 rodents and 13 species of the order Eulipotyphla. Our study shows that the facets of biodiversity are spatially incongruent. Net primary productivity has a positive effect on the three facets for most groups, while the effect of the NDVI is negative for TD and PD in most groups. AMT temperature and AP have negative effects on FD and PD, whereas TD is dependent on the species range scale. The human influence index effect on TD and PD also depends on the species range scale. These findings provide robust evidence that the ecological drivers of biodiversity differ among different biotas and different range scales, and future research should use multifacet approach to determine biodiversity conservation strategies.

## Graphical abstract

We investigate elevational patterns of three diversity metrics for small mammals and relate them to climate, productivity, vegetation, and human impact. We find ecological distinct groups of small mammals were unevenly affected by environmental and anthropogenic factors.

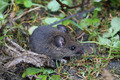

## 1 INTRODUCTION

Understanding the mechanisms shaping the distribution of biodiversity is a major goal in macroecology and conservation biogeography (Gaston, 2000). Biological community studies have usually used species richness (i.e., the total number of species in a specific area) to describe species diversity (Pavoine & Bonsall, 2011). Species are considered equally distinct when using species richness to measure biodiversity, providing little or no information about the phylogenetic relatedness of the species in a community and ecosystem functions that they perform (Tscharntke et al., 2012). Phylogenetic diversity (PD) and functional diversity (FD) have been suggested as alternative metrics with which to consider evolutionary history and species‐specific functional traits (Diaz et al., 2013) and may therefore facilitate a deep understanding of community assembly mechanisms (Safi et al., 2011).

Abiotic factors can affect species composition in a community by limiting the species to those who have specific functional traits necessary to live in these abiotic conditions (Graham, Parra, Rahbek, & McGuire, 2009; Webb, 2000). These abiotic conditions may act as a filter that prevents the establishment of species that have disfavored traits (Weiher, Clarke, & Keddy, 1998). Thus, FD is preferred over classic species diversity metrics because it is a strong indicator of which functional trait guilds can coexist in a community (Violle et al., 2007). In addition, FD reflects ecosystem functioning (Song, Wang, Li, & Zhou, 2014) and community assembly rules (Cornwell & Ackerly, 2009) and has implications for biodiversity conservation (Thakur & Chawla, 2019). On the other hand, PD reflects species evolutionary history and can be used as a measurement of species evolutionary differences in a community (Faith, 1992). Thus, the conservation of PD is the protection of evolutionary resources and provides a better understanding of the effects of evolutionary history on species interactions and abundance (Zhou et al., 2018). When closely related species have similar ecological strategies and their common ancestry within the community serves as a surrogate for species ecological similarity, one may expect the PD and FD to have a positive correlation based on phylogenetic niche conservatism (Wiens & Graham, 2005). Although PD can serve as a good surrogate for FD if target traits have evolved to follow the common ancestor's pattern, this is not always the case (Prinzing et al., 2008). In strong competitive environments, where species quickly diverge under adaptive radiation, the PD and FD may have no relationship or a negative relationship (Cachera & Le Loc'h, 2017). Therefore, it is better to calculate FD by measured trait data (Cadotte, Albert, & Walker, 2013). Increased TD can lead to more FD and PD because more species represent more functions and lineages. However, the relationship between the two is not always the same. Two assemblages that have the same TD may have different PD and FD because of differences in evolutionary histories and functional redundancy (Arnan, Arcoverde, Pie, Ribeiro‐Neto, & Leal, 2018). For example, after human land use, species succession may lead to communities with the same TD but very different FD and PD (Knapp et al., 2012). Recently, more research has focused on FD and PD to overcome the limitations of TD metrics for various taxa, such as mammals (Safi et al., 2011), insects (Arnan, Cerda, & Retana, 2015), and birds (Graham et al., 2009). Thus, a combination of different diversity indices is needed because it not only better addresses trends in biodiversity (Jarzyna & Jetz, 2016) but also gives a chance to have a high level of comprehension of the influences of species evolutionary distinctiveness, dispersal, and competitive ability on community assembly patterns (Belinchón, Hemrová, & Münzbergová, 2019; Violle et al., 2007).

Mountain ranges have exceptionally high biodiversity and therefore attract massive research on mountain elevation patterns (Quintero & Jetz, 2018). Mountain regions encompassing steep environmental gradients in small geographic areas are outstanding natural laboratories for biodiversity studies because many environmental factors have correlations with elevation, such as temperature (Wu et al., 2013), productivity (Ramirez‐Bautista & Williams, 2019), and anthropogenic disturbance (Santillán et al., 2020), which exert effects on biological communities (Körner, 2007). Although elevational biodiversity in TD has been investigated extensively, other key components, such as FD and PD, are still poorly tested compared with TD patterns (Burgio et al., 2019; Lopez‐Angulo et al., 2020). Karst mountains are very special landscapes shaped by rainfall and groundwater acting on carbonate bedrock. They are widely distributed over approximately 15%–20% of the Earth's ice‐free land surface, with the largest continuous area of approximately 0.51 million km^2^ located in Southwest China. Karst mountains are highly sensitive and vulnerable, making biodiversity conservation important (Jiang, Lian, & Qin, 2014). The karst mountain area in Southwest China is one of 25 global biodiversity hotspots because of its unique biome. Although 19 national nature reserves have been established in China karst mountain areas (Liu, Williams, & Tan, 2017), there is still a lack of comprehensive studies on the biodiversity pattern and environmental impacts on biodiversity in this hotspot. Biodiversity conservation in such areas not only faces pressure from economic development but also fragile geological environments and high habitat heterogeneity (Tong et al., 2016).

**Figure 1:**
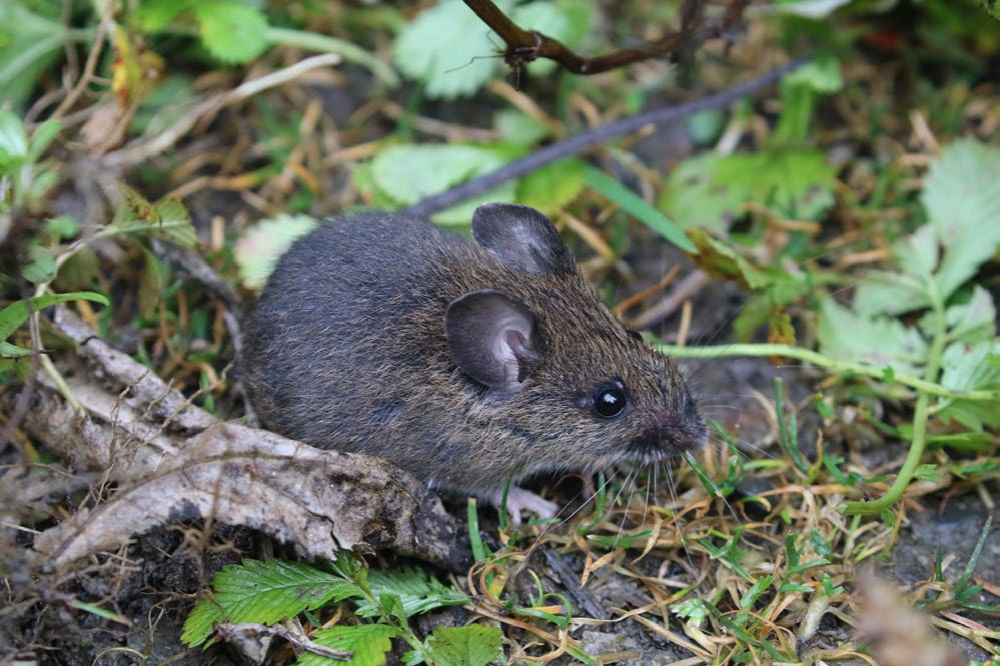
A representative rodent species (*Apodemus draco*) in the Wuling Mountain Area (photograph taken by Nan Yang)

Small mammals, such as rodents and insectivores, are the most diverse group of mammalian species, showing a wide range of sizes, behaviors, and niche uses (Nowak & Walker, 1999). They play key roles in trophic and food web dynamics, including important ecological roles in mountain ecosystems such as arthropod predators (Carvalho, Fernandez, & Nessimian, 2005), seed predators and dispersers (Martin‐Regalado, Briones‐Salas, Lavariega, & Moreno, 2019), as ecosystem engineers in soil aeration and nutrient mixing through their burrowing (Zhang, Zhang, & Liu, 2003), and as a resource for organisms in higher trophic levels (Wright, Gompper, & DeLeon, 1994). These taxa are particularly suitable for examining elevation patterns because they are commonly found along mountain slopes, have higher speciation rates and higher species turnover between habitats compared with larger mammals (Lopez et al., 2016). Indeed, a rodent species was recently discovered to be the world's highest dwelling mammal (Storz et al., 2020). In this context, small mammal communities are among the best models for disentangling the interplay between the facets of biodiversity. However, little attention has been paid to integrating all three facets of biodiversity in small mammals (Figure 1).

**Figure 2:**
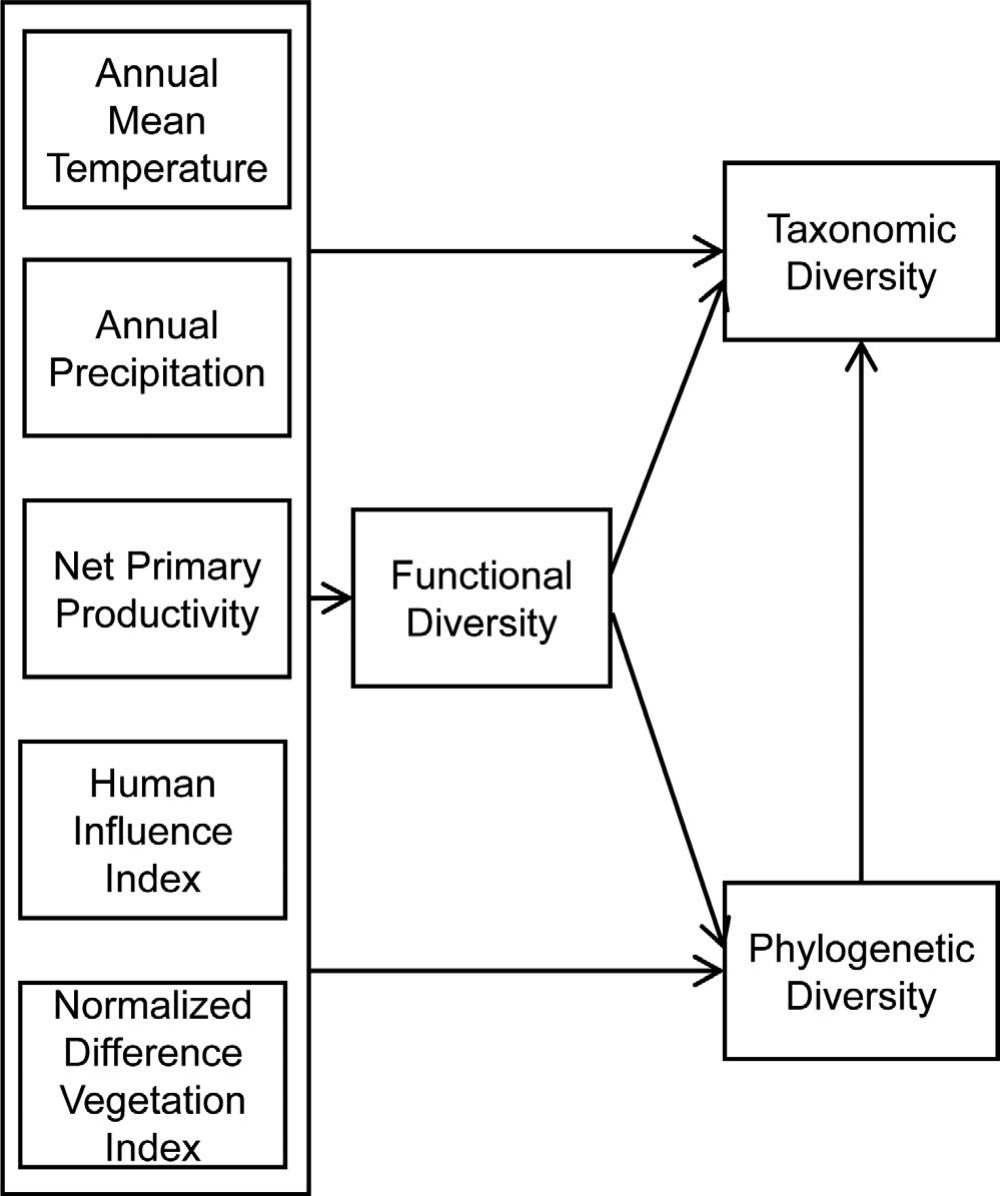
Hypothesized causal relationships of the structural equation model including the effects of climate factors (annual mean temperature and annual precipitation), vegetation structure (normalized difference vegetation index), productivity (net primary productivity), and human disturbance (human influence index) on the three diversity facets (taxonomic diversity, phylogenetic diversity, and functional diversity). Arrows represent direct causal relationships

Environmental gradient‐related factors such as temperature, precipitation, productivity, vegetation structure, and anthropogenic disturbances play a key role in small mammal biodiversity (McCain & Colwell, 2011). To discern the relationship between these biodiversity facets (TD, FD, and PD) and environmental factors, we tested a theoretical framework (Figure 2) using structural equation modeling (SEM). First, in natural communities, all of the factors may have a direct impact on TD, PD, and FD. Second, species functional traits may be indicative of the presence of lineages or species (Le Bagousse‐Pinguet et al., 2017). However, if some critical functional traits that cannot be measured are not included in the FD calculation, FD may not explain all of the variation in PD and TD (Hurtado et al., 2019). Here, we focus on the elevation patterns of small mammal biodiversity across the Wuling Mountains in Southwest China and aim to prove the following predictions: (a) The TD, PD, and FD show different elevation patterns across the mountains, given that these indices consider different biodiversity aspects; (b) because of the different ecological roles of environmental factors, we expect these different ecological drivers (climate, vegetation, and human disturbance) to be related to these facets of biodiversity in different ways; and (c) different ecological drivers have different effects on different taxa and at different range scales, considering their different environmental adaptability.

## 2 METHODS

### 2.1 Study area

**Figure 3:**
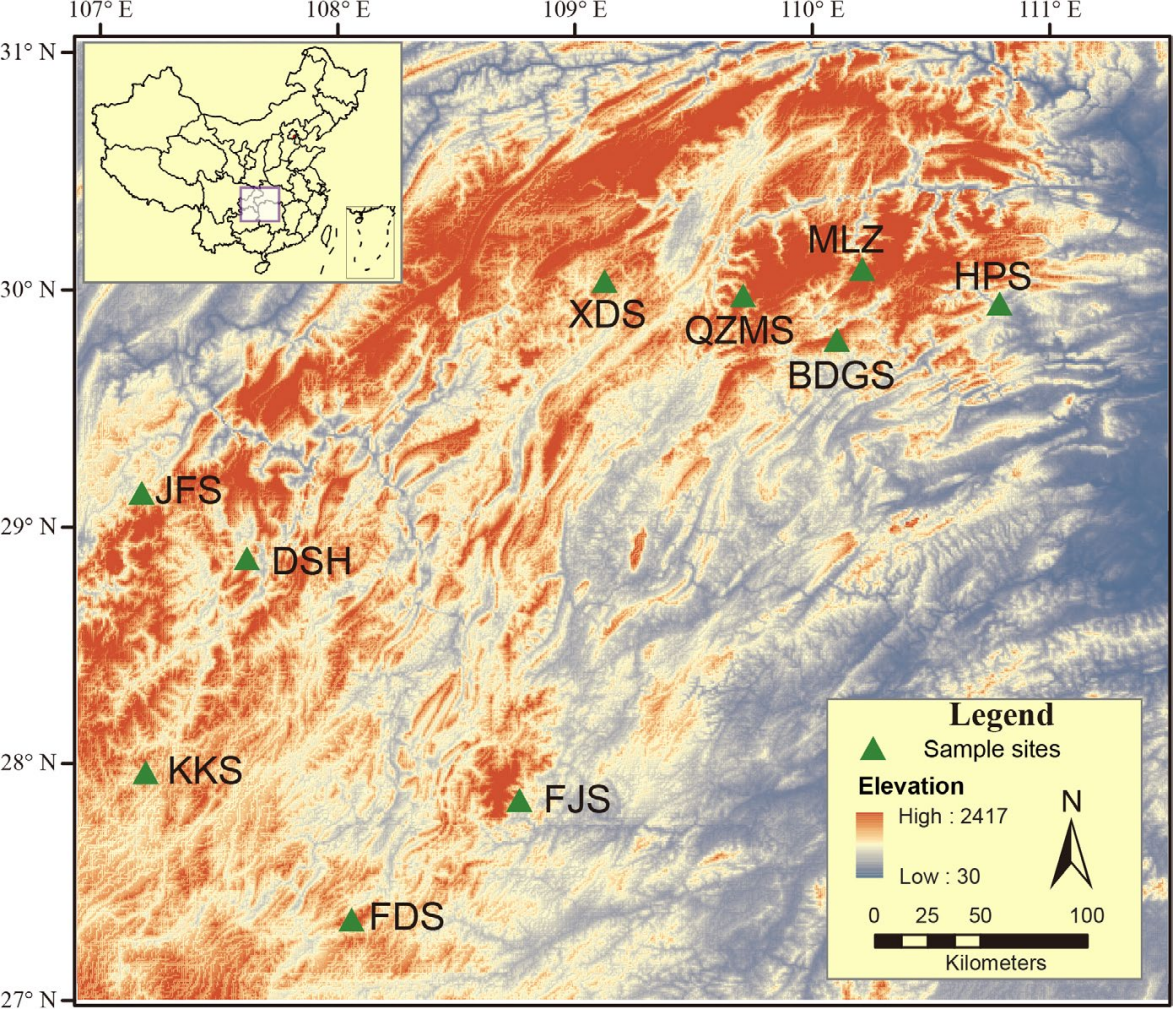
Distribution of sampling sites in the Wuling Mountains Area

The Wuling Mountains Area is located at the junction of the Oriental and Palearctic Regions in Southwest China, stretching across Chongqing, Hunan, Hubei, and Guizhou provinces (N 27°28′–33°05′, E 107°02′–111°33′) (Figure 3). The area is one of 200 important ecological areas of global priority protection and is the core area of the ecological zone of the subtropical broad‐leaved forest in China (Tang, Wang, Zheng, & Fang, 2006). The mountains are of karst geomorphology and form a key international biodiversity zone because of their diverse flora and fauna.

### 2.2 Small mammal data collection

From 2014 to 2019, our research group conducted eight fieldwork studies on Mt. Fanjing, Mt. Foding, Mt. Badagong, Mt. Huping, Mt. Qizimei, Mt. Jinfo, Mt. Dashahe, and Mt. Kuankuoshui, which are national nature reserves belonging to the Wuling Mountains. We collected small mammals along an elevation gradient at these localities. Historical studies on small mammals from Mt. Xingdou and Mt. Mulinzi were also incorporated into our analyses. In the eight mountains that our group investigated, standardized trapping procedures were used to collect small mammals. In every elevational gradient, 400–600 snap traps were used to catch small mammals. The number of traps varied among habitats, and the distance between two traps was approximately 3 m. We placed peanuts on the traps to attract small mammals and checked the traps every morning. Continuous trapping at each site occurred for at least six days until the species accumulation curves became asymptotic. For the two mountains studied by a previous scientific survey, similar sampling methods were used to collect small mammals. Range interpolation was performed for each species, and this method assumes species to be present or potentially present between their upper and lower recorded elevational bands (Wu et al., 2013). This approach is widely used in elevation research, and this interpolated presence–absence dataset was used for statistical analysis. We further divided the small mammals into Rodentia and Eulipotyphla species according to their order and into “narrow‐range” and “broad‐range” species (Hu et al., 2017). Rodentia and Eulipotyphla species were defined by their order. The narrow‐range species group included species with an elevation range less than 900 m, while the broad‐range species were those with an elevation range larger than 900 m. Because 900 m is one‐half of the elevation difference in the Wuling Mountains, the median size is commonly used to separate mountain species range scales (Hu et al., 2017; Pan et al., 2019). We separate the whole mountain range into nine gradients, and every elevation range is an interval of 200 m because this 200‐m interval is better able to capture the degree of variability in mammal diversity than other interval sizes (Chen, He, Cheng, Khanal, & Jiang, 2017; Hu et al., 2017).

### 2.3 Trait data

Nine commonly used small mammal functional traits considered drivers of biodiversity and ecosystem function relationships were selected for analysis: two binary traits (diet and habitat), two categorical traits (foraging stratum and daily activity), and five continuous traits (ear length, hind foot length, tail length, body length, and body mass). For the morphological traits, we measured the specimens deposited at the Institution of Zoology, Chinese Academy of Sciences, Beijing, China (IOZCAS), and other traits were extracted from Elton Traits 1.0 (Table S4) (Wilman et al., 2014).

### 2.4 Phylogenetic analyses and phylogenetic signal

The cytochrome *b* (Cyt*b*) gene was used to reconstruct a phylogenetic tree (Figure S2), and sequences were obtained from GenBank. We first aligned the Cyt*b* gene by the MUSCLE algorithm in MEGA X (Kumar, Stecher, Li, Knyaz, & Tamura, 2018), and then, the best‐fit nucleotide substitution model was selected by jModelTest under the Akaike information criterion (AIC). The last step was using MrBayes software to calculate species phylogenetic relationships through Bayesian inference, and Markov chain Monte Carlo (MCMC) analyses were calculated with 20 million generations for posterior distributions. The first 25% of the Markov chain samples were discarded as burn‐in, and the remaining samples were used to generate a majority rule consensus tree. Then, the trees created by MrBayes were exported in FIGTREE software. To identify possible differences in patterns of functional and phylogenetic structure, we quantified the phylogenetic signal (i.e., the similarity of species functional traits and their phylogenetic relatedness). If a significant phylogenetic signal was detected, it means that closely related species have higher similarity than expected by chance (Revell, Harmon, & Collar, 2008). For continuous traits, the phylogenetic signal was quantified using the *K* statistic. *K* ≥ 1 suggests the presence of a phylogenetic signal. If *K* approaches zero, there is a weak phylogenetic signal (Blomberg, Garland, & Ives, 2003). For binary traits, the *D* statistic was used to quantify the phylogenetic signal. If *D* approaches 0, then there is conserved trait evolution. *D* < 0 suggests trait clustering. A value of *D* ≥ 1 indicates no phylogenetic signal or a trait that is overdispersed on the phylogenetic tree (Fritz & Purvis, 2010). The *D* statistic and *K* statistic were calculated by using the “phylo.d” and “multiPhylosignal” functions in the “caper” and “picante” packages, respectively (Kembel et al., 2010; Orme, Freckleton, Thomas, & Petzoldt, 2013). For categorical traits, we use a new analytical procedure to incorporate evolutionary models into Mantel (EM‐Mantel) tests. This test has higher statistical power and lower type I error rates. We use the original R code provided by Debastiani and Duarte (2017) and ran the evolutionary model 999 times to obtain the null distributions of the Mantel coefficient for the conventional Mantel and EM‐Mantel tests, respectively (Debastiani & Duarte, 2017).

### 2.5 Environmental data

To explore the effects of environmental factors on biodiversity, we extracted annual mean temperature (AMT) and annual precipitation (AP) data in 30‐arc second (~880 m)‐resolution grids for each elevation range from WorldClim (Fick & Hijmans, 2017). In addition, we extracted normalized difference vegetation index (NDVI) and net primary productivity (NPP) data from the China Resource Discipline Innovation Platform and human influence index (HII) data from the Socioeconomic Data and Applications Center. All the data were extracted using the “raster” package in R software (Hijmans & van Etten, ).

### 2.6 Diversity metrics

In this study, we calculated three distinct facets of biodiversity in the small mammal communities: TD, FD, and PD. SR was used as a measure of TD, and mean pairwise distances (MPDs) were chosen to estimate FD and PD. Functional distances described the functional dissimilarity between all species pairs within a community (Sandrine Pavoine, Vallet, Dufour, Gachet, & Daniel, 2009) and were calculated using the function “daisy” in the “cluster” package (Maechler, 2019). The phylogenetic pairwise dissimilarity was calculated in the “ape” package by the “cophenetic” function (Paradis, Claude, & Strimmer, 2004). Calculation of MPDs was performed using the “melodic” function (de Bello, Carmona, Lepš, Szava‐Kovats, & Pärtel, 2016). We also downloaded every species’ evolutionary distinctiveness (ED) value, which is based on the fair‐proportion index (Isaac, Turvey, Collen, Waterman, & Baillie, 2007) from the EDGE website (http://www.edgeofexistence.org) (Zoological Society of London, 2008), and used these scores to calculate community evolutionary distinctiveness (CED), these ED values were chosen from public database because they can reflect ED for all mammals in Wuling Mountains. The CED was the average ED score from all the species in an elevation range.

### 2.7 SEM analyses

The relationships between the selected environmental predictors and biodiversity metrics were analyzed using an SEM approach. Theoretical models were proposed to show causal relationships between biodiversity metrics and environmental factors (Figure 2). We expected that environmental variables would directly modify FD, PD and TD. All the environmental factors could also have an effect on the FD of the small mammal community and, afterward, the PD and TD. In addition, the relationship between PD and TD was proposed.

Before modeling, logarithmic transformations and standardization of each variable were applied to allow direct comparisons of the path coefficients in the SEM models. The maximum‐likelihood method was used to estimate path coefficients because of its robustness against certain multinormality deviations (Shipley, 2016). A multi‐index approach was used to assess model quality: the comparative fit index (CFI > 0.95; higher values indicate good models), AIC (lower values indicate good models, subsequent metrics are the same standard), standardized root mean square residual (SRMR < 0.1), and root mean square error of approximation (RMSEA < 0.08). The SEM models were fit in the “lavaan” package (Rosseel, 2012).

## 3 RESULTS

### 3.1 Diversity record

**Figure 4:**
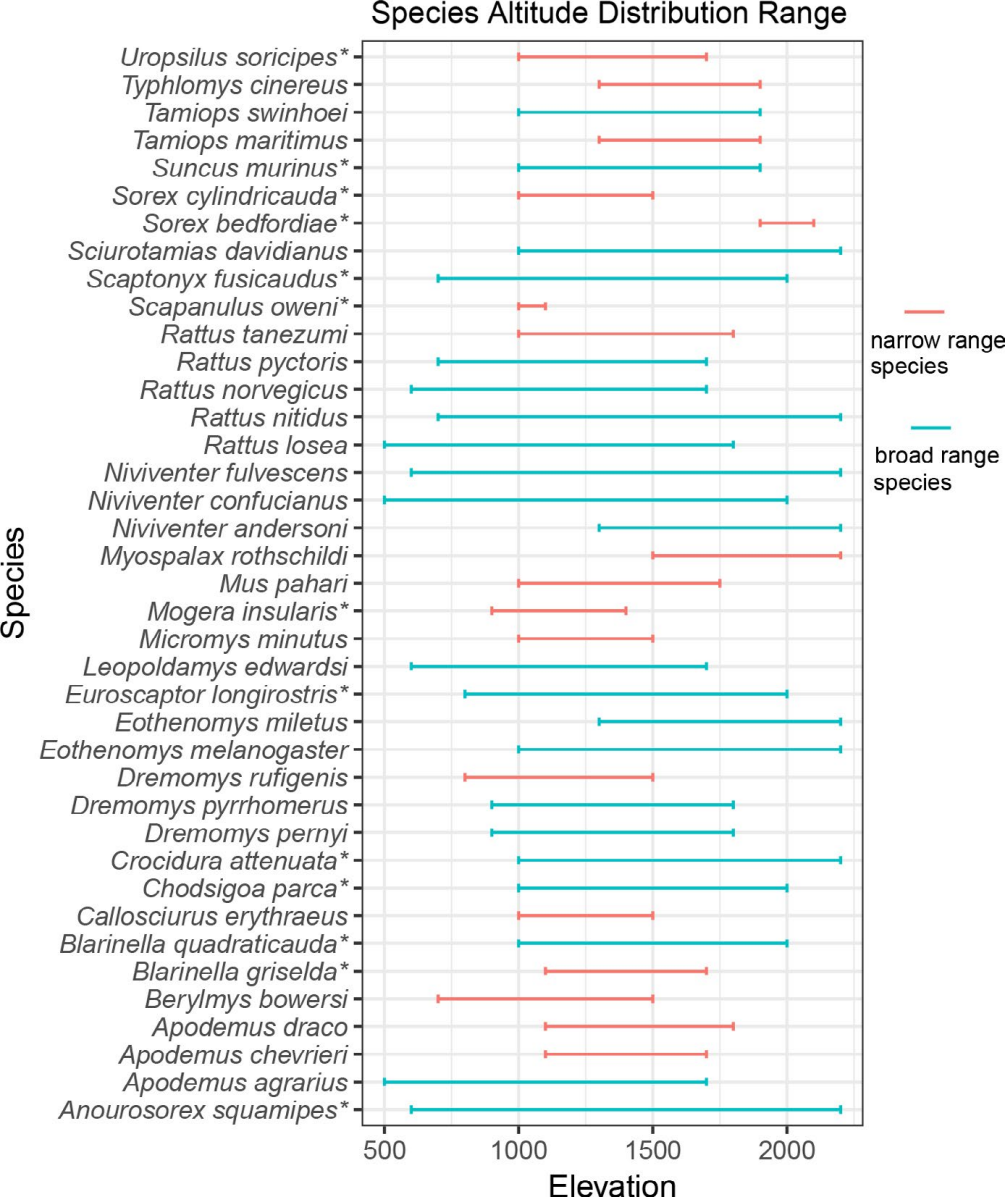
Elevational distribution ranges of small mammal species in the Wuling Mountains. Red lines are narrow‐range species, and green lines are broad‐range species. Species names with the “*” symbol are Eulipotyphla, and species names without a symbol are Rodentia

We recorded a total of 39 small mammal species in nine elevation ranges in the Wuling Mountains (Figure 4). These species belonged to 26 species in the order of Rodentia and 13 species in the order of Eulipotyphla. According to their range scale, they were grouped into 22 broad‐range species and 17 narrow‐range species.

### 3.2 Elevation patterns of variables and biodiversity

**Figure 5:**
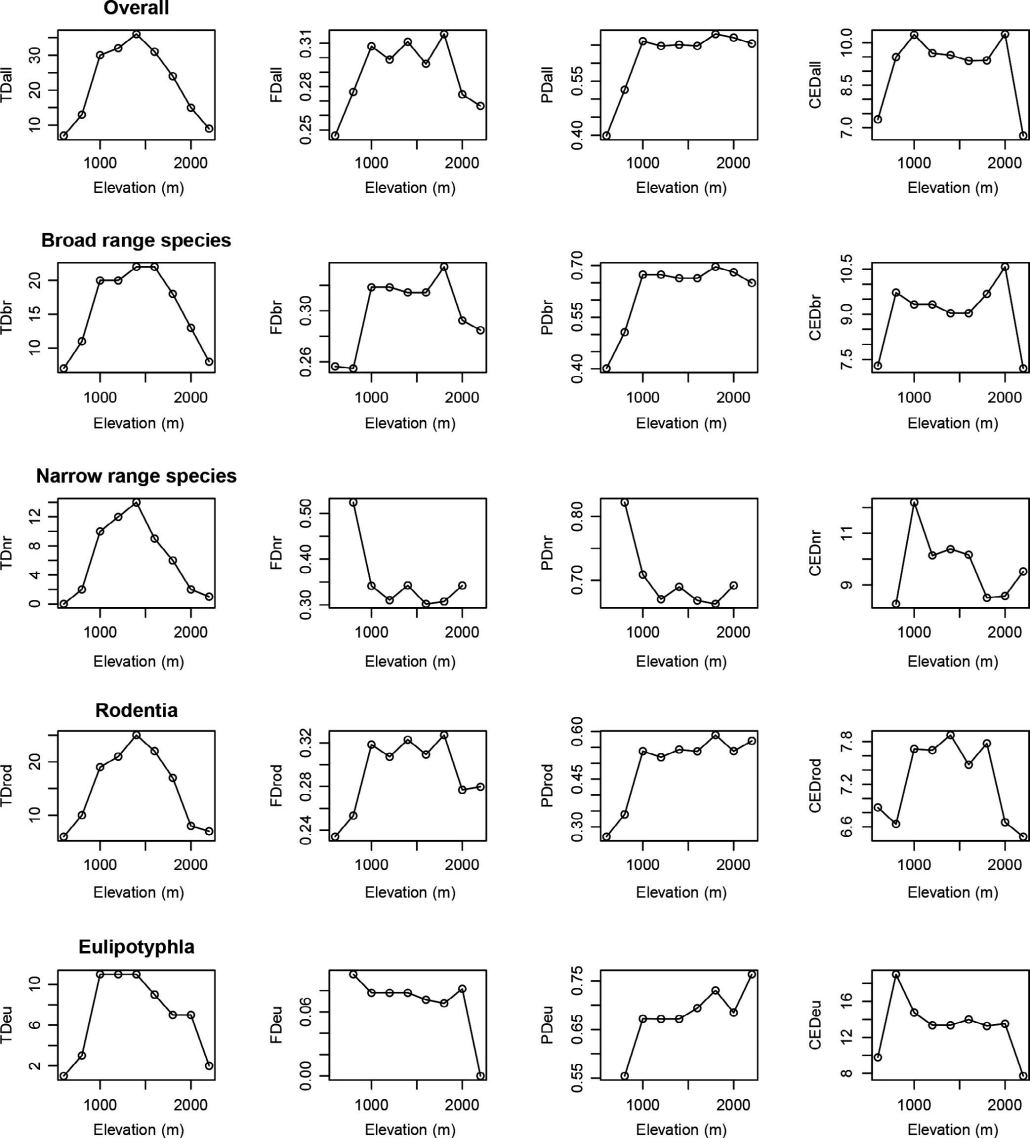
Elevational patterns of biodiversity facets in the Wuling Mountains

Different environmental factors had different elevation patterns (Figure S1). AMT decreased with increasing elevation. AP decreased at low elevations and increased with elevation. NPP and NDVI increased along the elevation gradient and showed a decrease at the highest elevation. HII decreased from low elevations to middle elevations and then increased at high elevations. Elevation patterns of biodiversity differed among the biodiversity dimensions (Figure 5). Mid‐elevations have higher CED than the lower and higher elevations (Figure 5). For the TD‐elevation relationship, all species, narrow‐ and broad‐range species, and Rodentia and Eulipotyphla species all peaked at middle elevations, showing a hump‐shaped pattern. PD showed an inconsistent trend across groups. The PD of all species, broad‐range species, Rodentia species, and Eulipotyphla species increased from low elevations to 1,000 m and then varied at middle to high elevations, but the PD of narrow‐range species decreased from low elevations to 1,200 m and maintained a low value from middle to high elevations. FD also showed an inconsistent trend across groups. The FD of all species, broad‐range species, and Rodentia species displayed approximately asymmetrical hump‐shaped patterns, narrow‐range species showed a pattern similar to that for PD, and Eulipotyphla had a very low value at all elevations.

### 3.3 Phylogenetic signals

Functional trait data of the 39 small mammal species are presented in Table S4. The *K* values of body length and body mass were close to 1 (*p* value = .001). The *K* values of ear length, hind foot length, and tail length were greater than 1, which proved that these functional traits had significant phylogenetic signals. All the *D* statistics demonstrated high clustering for the binary traits (*D* < 0 and *p* = 0). Although categorical traits had a significantly positive Mantel correlation (*p* < .01), the positive correlation showed no significant differences from neutral model simulations (forage stratum: *p* = .1235 and activity time: *p* = .0939) (Table S5).

### 3.4 SEM

**Figure 6:**
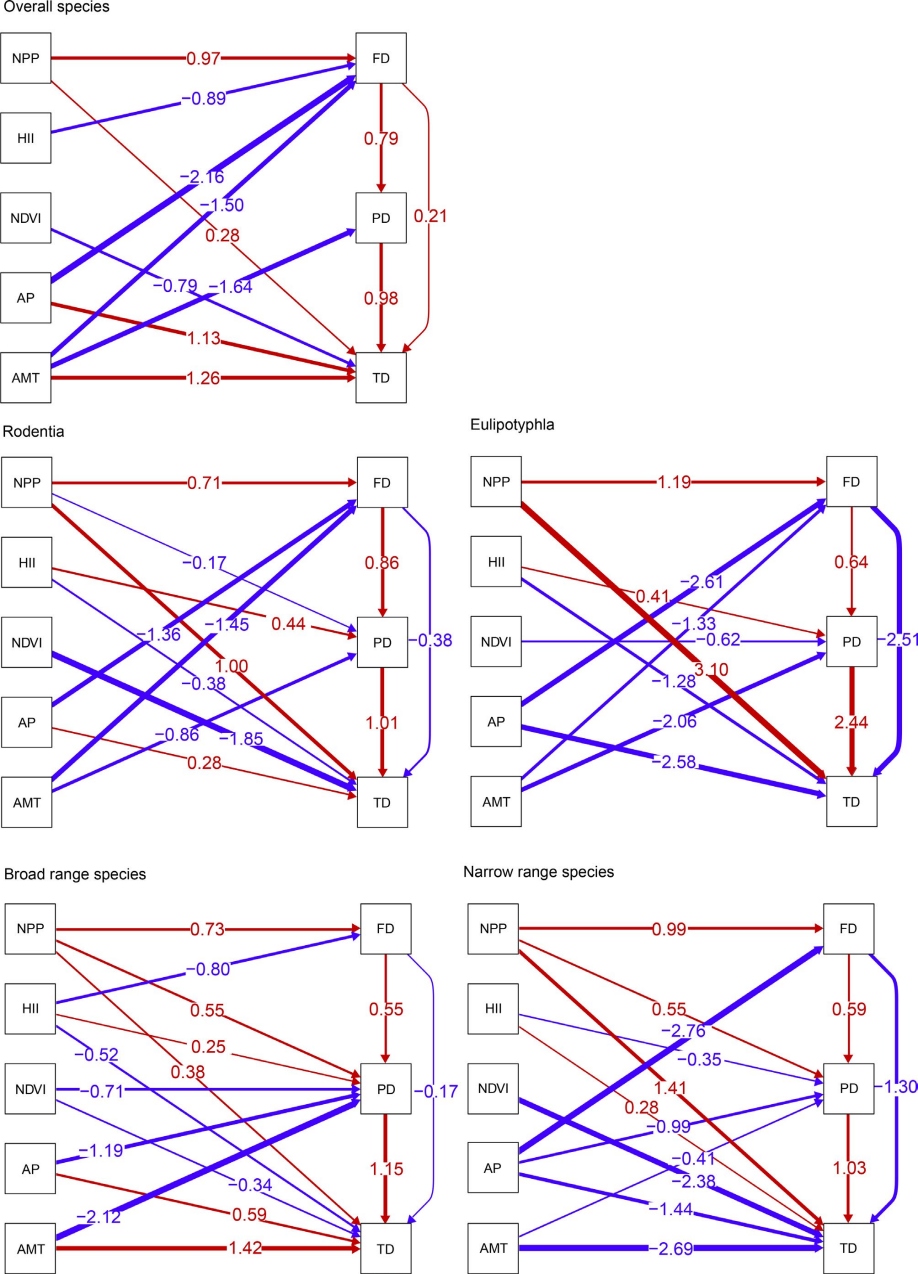
SEM models in the Wuling Mountains Area for overall species, broad‐range species, narrow‐range species, Rodentia, and Eulipotyphla. Arrows represent significant path coefficients (at *p* < .05). Blue arrows represent negative paths, and red arrows represent positive paths. Line thickness is proportional to the standardized coefficient strength. Path coefficients that were not significantly different from zero were omitted

PD was positively correlated with FD and TD for all groups. FD was negatively correlated with TD in all groups of species but not for all species considered together. Different facets of biodiversity in different groups were affected by different factors. For TD, climate variables including AP and AMT had a strong effect on all species and broad‐ and narrow‐range species, but taxa with different range scales showed different responses. Groups with different range scales also showed different responses to the HII. NPP and the NDVI had strong but opposite effects on most groups. For PD, climate variables and the NDVI were important determinants in most groups. The HII and NPP mainly affected taxa with different range scales and belonging to different orders. FD showed a negative relationship with climate variables and the HII for most of the groups, but NPP was positively correlated with FD for all the groups (Figure 6).

## 4 DISCUSSION

### 4.1 The elevation pattern of biodiversity facets

In this study, we deconstructed the biodiversity gradient by taxonomic group and distribution range scale and assessed the drivers of biodiversity in the Wuling Mountains. In general, the elevation patterns of TD for all small mammal groups (all, Rodentia, Eulipotyphla, broad‐range and narrow‐range species) were hump‐shaped, consistent with the findings of most previous elevation diversity studies of small mammals (Hu et al., 2017; Rowe, 2009; Shuai, Ren, Yan, Song, & Zeng, 2017; Wen et al., 2018). Hump‐shaped patterns seem to be the dominant patterns along mountain slopes. However, spatial mismatch is often found in the FD and PD of mammals (Martin‐Regalado et al., 2019). The FD of all species, broad‐range species, and rodents also had a mid‐elevation peak, similar to the trend observed for small mammal TD. The decay in FD of narrow‐range species is attributable to the loss of distinctive functional traits with elevation. In contrast, the higher FD of Eulipotyphla at high elevations may represent the following mechanisms: a few species adapted to high elevations by developing extreme traits, or there was a loss of functionally redundant species at higher elevations, resulting in the maintenance of species that have extreme traits (Montaño‐Centellas, McCain, Loiselle, & Grytnes, 2019). Most groups, with the exception of narrow‐range species that live at middle to high elevations, showed high PD at high elevations. This result may be explained by the strong anthropogenic disturbance at lower elevations acting as a significant filter, removing specialized taxa and phylogenetically distant groups and leaving more generalists and closely related species (Arnan et al., 2018).

### 4.2 Effects of environmental factors on TD

Ecologists have long recognized that climate factors have a major impact on species diversity and composition, but the underlying mechanisms remain divergent. There are two main hypotheses regarding these mechanisms: the ambient energy hypothesis and the productivity hypothesis (Hawkins et al., 2003). The microclimates in small mammal caves have scant resemblance with the outside world. Small mammals can expose themselves to outside environment for relatively short periods of time to avoid the temperature effects; thus, temperature alone is a poor predictive factor for small mammals (Speakman, 1997). We should expect a positive association between primary productivity and TD if the productivity hypothesis is correct (Shuai et al., 2017). Our results partially support this hypothesis: NPP, a productive energy parameter, exhibited a strong positive relationship with TD for all groups, and AP was positively related to TD for most of the groups (all species, broad‐range species, and Rodentia species). Changes in precipitation patterns provide a chance for high‐elevation species to expand their population (Duclos, DeLuca, & King, 2019). The negative effects of precipitation that we found for narrow‐range species and Eulipotyphla species are in concordance with the findings of previous studies showing that heavy rain events may have negative effects on many taxa (Aizen et al. 2003) potentially because of associated foraging restrictions (Boyle, Norris, & Guglielmo, 2010).

Habitat fragmentation caused by human activity and human population growth are the main causes of wildlife species extinction (McKee, Chambers, & Guseman, 2013). Our results show that human influence had a negative correlation with TD in the Wuling Mountains for most of the groups, similar to findings elsewhere in China (Pautasso, 2007). Humans have resided at low elevations in the Wuling Mountains Area for a long time (Li & Zhang, 2017). The presence of farms and villages at low elevations results in strong anthropogenic disturbance, which may have eliminated many small mammals that are not suitable for this artificial environment. At middle to high elevations, the Chinese government built many protection areas a few decades ago (Liu et al., 2017), and these reserves play an important role in protecting small mammals. However, at the top of the mountains, there are still many temples (Zhang, 2014), which attract many tourists. This may explain why the HII has increased at elevations above 2,000 m, while the species richness has decreased. Although some studies have used the NDVI as a substitute for NPP, we found that the NDVI was also a more suitable proxy than NPP for vegetation structure (Flores‐Manzanero, Luna‐Barcenas, Dyer, & Vazquez‐Dominguez, 2019). Global vertebrate diversity has been shown to have a significant correlation with vegetation structure (Kissling, Sekercioglu, & Jetz, 2012). Here, we found a negative correlation between vegetation structure and TD. Complex vegetation structures are considered to be a relevant environmental filter, eliminating some species that cannot adapt to complex environments within the community (Devictor et al., 2010).

### 4.3 Effects of environmental factors on FD and PD

Phylogenetic signal tests found strong correlations between functional traits and phylogenetic relatedness, meaning that closely related species have more similar functional traits. The PD and FD indices responded in the same way to temperature and precipitation. The correlation between PD and temperature has different mechanisms in different regions. A negative relationship between higher temperature and PD has been detected in some tropical areas. The mechanism underlying this phenomenon suggests that temperature has a primary effect on the acceleration of mutation and metabolic rates, and thus, the rate of speciation can be more rapid (Barreto, Graham, Rangel, & Belmaker, 2019). Our results showed a negative relationship in the Wuling Mountains, supporting the notion that the acceleration of speciation rates is the predominant factor for small mammals in this karst area. Other studies found that FD is positively correlated with temperature or precipitation (Martin‐Regalado et al., 2019); however, in our study, a negative correlation was found. The mechanism behind this phenomenon could be that environmental conditions act as a filter. This scenario filters out specific traits, and species coexisting in a certain community share this specific trait with more similarity than would be expected by chance (Zobel, 1997). More productivity may increase FD and PD for small mammals by providing more available niche space, and species that have complementary ecological strategies can survive in competitive exclusion (Brun et al., 2019). The difference in the PD‐HII relationship between broad‐range and narrow‐range species suggests that these groups of species respond differently to anthropogenic disturbances due to the greater acclimatization ability of broad‐range species compared with narrow‐range species in disturbed habitats. Previous studies proved the expectation that disturbance can be a filter, and only a few related lineages can colonize and adapt to anthropogenically disturbed conditions (Helmus et al., 2010). The traits of co‐occurring species can be limited by anthropogenically disturbed environments, and thus, the amount of functional variation in a community can also be constrained. For instance, for a community that is disturbed by human activity, species richness does not change because, during disturbance, some species may be lost, but broad‐range species with generalist traits can enter these disturbed areas and colonize successfully (Graham & Blake, 2001; Renjifo, 2001). This change in abundance pattern likely explains why strong anthropogenic disturbance greatly reduces FD (Tinoco, Santillan, & Graham, 2018).

## 5 CONCLUSION

In conclusion, our findings revealed spatial incongruence among taxonomic, functional, and phylogenetic diversity for small mammals along elevation gradients in karst mountains. The environmental factors affecting different facets of biodiversity differ between taxa and range scales. We also emphasize that our integrative approach based on a multirange scale and multitaxa perspective has a better understanding in the interactions between ecological parameters and biodiversity metrics. Future methodological improvements should focus on using more kinds of baits to attract rare species and increasing the distance between the adjacent traps to cover a larger habitat for small mammals. Our integrated approach can also be applied to other communities, which have different climate conditions and contrasting evolutionary histories to help define protected areas.

## 6 DATA ACCESSIBILITY STATEMENT

Data are available from the Dryad Digital Repository: https://doi.org/10.5061/dryad.m37pvmd0c

## CONFLICT OF INTEREST

None declared.

## AUTHOR CONTRIBUTION

**Jian Sun:** Conceptualization (lead); Data curation (equal); Formal analysis (lead); Methodology (equal); Software (lead); Writing‐original draft (lead); Writing‐review & editing (lead). **Zhixin Wen:** Data curation (equal); Methodology (equal); Writing‐review & editing (equal). **Anderson Feijó:** Data curation (equal); Formal analysis (equal); Methodology (equal); Software (equal); Writing‐review & editing (equal). **Jilong Cheng:** Data curation (equal); Resources (equal); Software (equal); Writing‐review & editing (equal). **Yanqun Wang:** Data curation (equal); Resources (equal). **Song Li:** Data curation (equal). **Deyan Ge:** Data curation (equal); Writing‐review & editing (equal). **Lin Xia:** Data curation (equal); Resources (equal). **Qisen Yang:** Conceptualization (equal); Data curation (equal); Funding acquisition (lead); Project administration (lead); Supervision (equal); Validation (equal); Writing‐review & editing (equal).

## Supporting information

Figure S1

Figure S2

Tables S1–S7
