# Supplementary figures and images for: Elevation patterns and critical environmental drivers of the taxonomic, functional, and phylogenetic diversity of small mammals in a karst mountain area
Source: Ecol Evol. 2020 Sep 9;10(19):10899–911. doi: 10.1002/ece3.6750 (PMC7548175; doi:10.1002/ece3.6750)

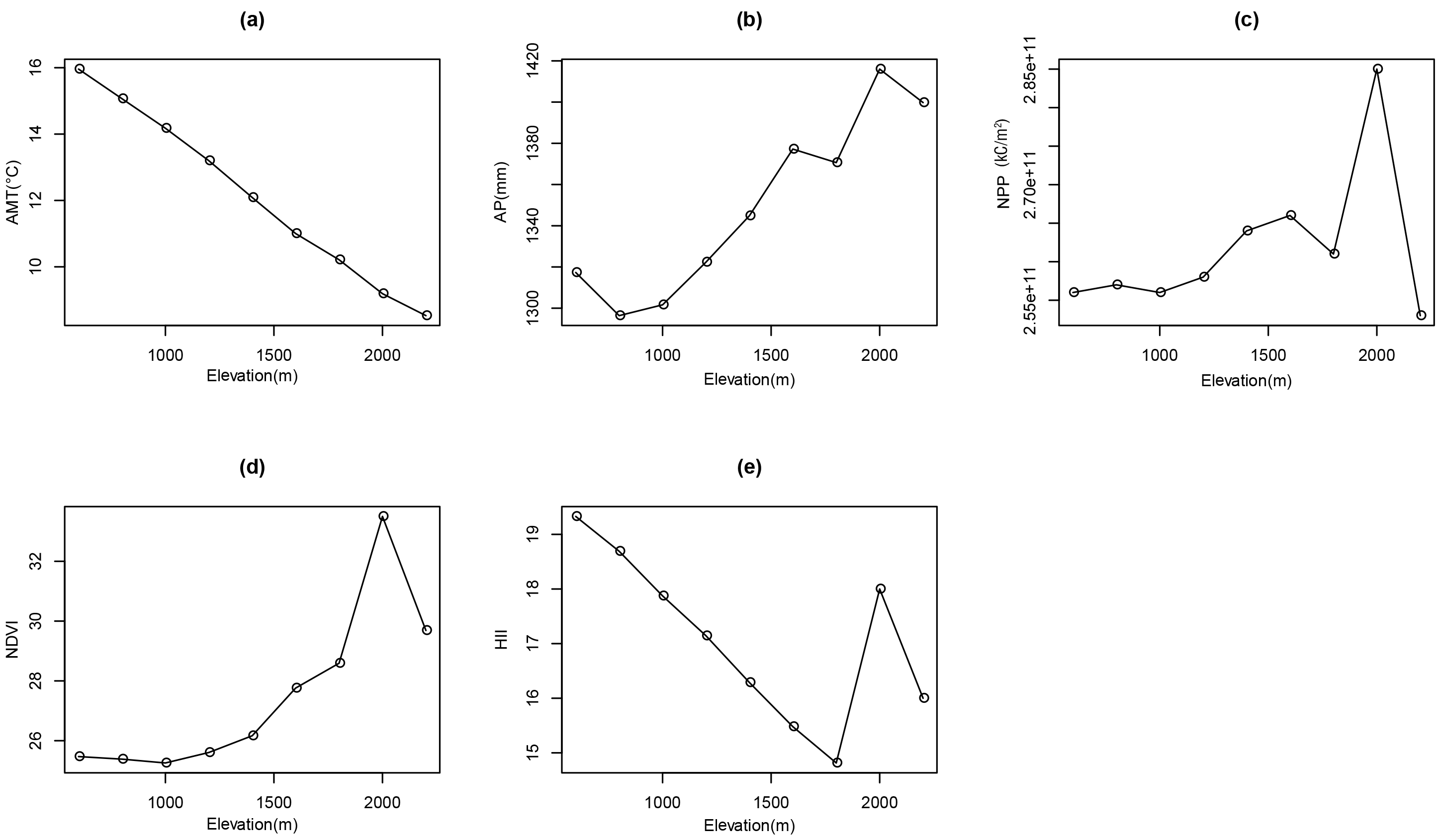

Supplement: Supplementary file 1 — Figure S1 [file ECE3-10-10899-s001.tif]

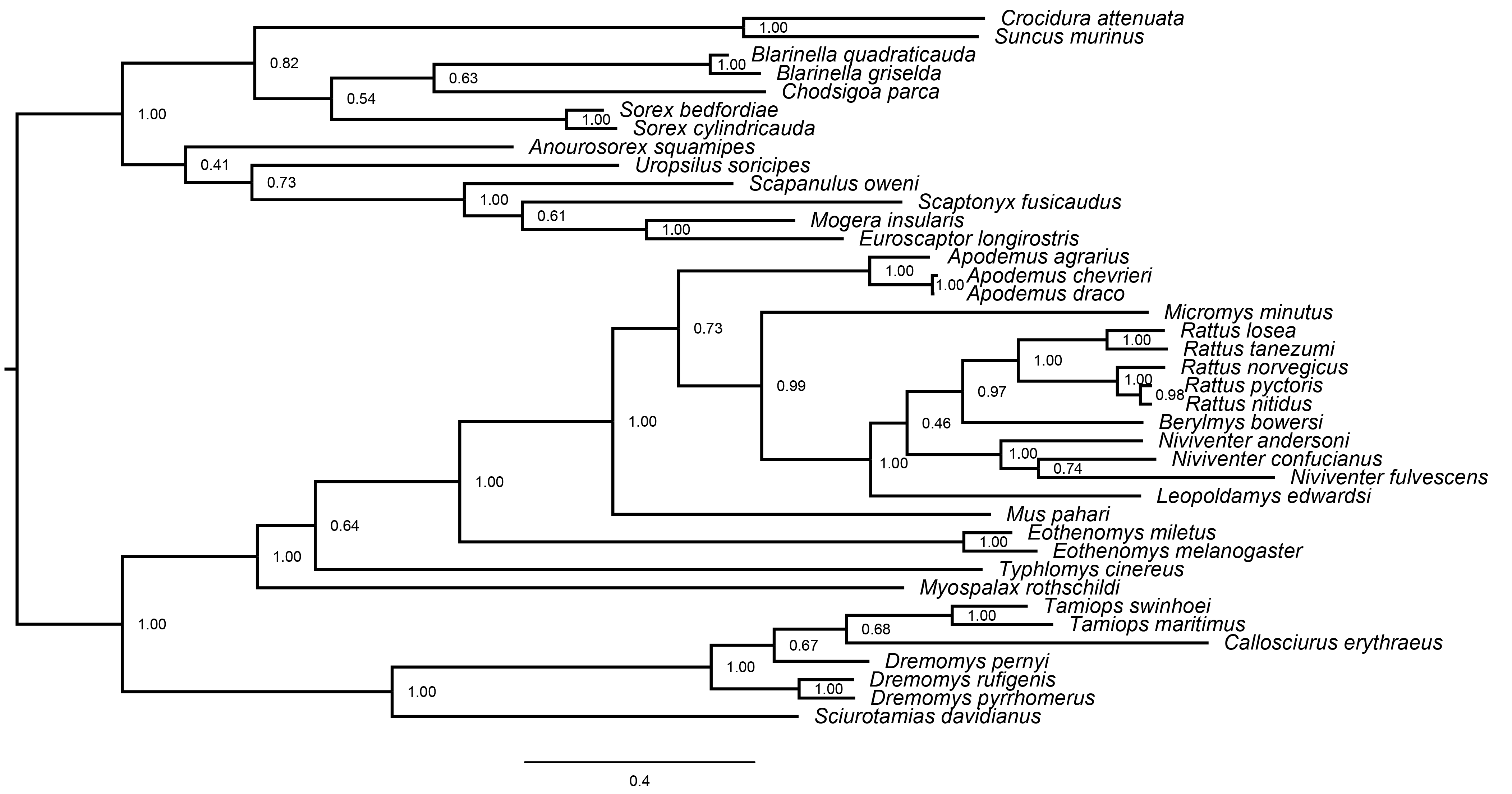

Supplement: Supplementary file 2 — Figure S2 [file ECE3-10-10899-s002.tif]
