# Supplementary material for: Elevation patterns and critical environmental drivers of the taxonomic, functional, and phylogenetic diversity of small mammals in a karst mountain area
Source: Ecol Evol. 2020 Sep 9;10(19):10899–911. doi: 10.1002/ece3.6750 (PMC7548175; doi:10.1002/ece3.6750)
Supplement: Supplementary file 3 — Tables S1–S7 [file ECE3-10-10899-s003.docx]

Supplementary material of the article “Elevation patterns and critical environmental drivers of the taxonomic, functional and phylogenetic diversity of small mammals in a karst mountain area”

Table S1 Sample sites in the Wuling mountain area.

| site | Mt. Huping | | | | Mt. Badagong | | | Mt. Foding | | | | Mt. Kuankuoshui | |
| --- | --- | --- | --- | --- | --- | --- | --- | --- | --- | --- | --- | --- | --- |
|  | 900m | 1300m | 1600m | 1800m | 600m | 1050m | 1450m | 900m | 1250m | 1500m | 1750m | 900m | 1400m |
| Anourosorex squamipes | 0 | 1 | 1 | 1 | 1 | 1 | 1 | 0 | 1 | 1 | 0 | 0 | 1 |
| Apodemus agrarius | 1 | 0 | 0 | 0 | 1 | 1 | 0 | 1 | 0 | 0 | 0 | 1 | 1 |
| Apodemus chevrieri | 0 | 0 | 0 | 0 | 0 | 0 | 0 | 0 | 0 | 0 | 1 | 0 | 0 |
| Apodemus draco | 0 | 1 | 1 | 0 | 0 | 0 | 1 | 0 | 0 | 0 | 0 | 0 | 0 |
| Berylmys bowersi | 0 | 0 | 0 | 0 | 0 | 0 | 0 | 0 | 0 | 0 | 0 | 0 | 1 |
| Blarinella griselda | 0 | 0 | 0 | 0 | 0 | 0 | 1 | 0 | 1 | 1 | 1 | 0 | 0 |
| Blarinella quadraticauda | 0 | 0 | 0 | 0 | 0 | 0 | 0 | 0 | 0 | 0 | 0 | 1 | 1 |
| Callosciurus erythraeus | 1 | 1 | 1 | 0 | 0 | 1 | 1 | 1 | 1 | 1 | 0 | 1 | 1 |
| Chodsigoa parca | 0 | 0 | 0 | 0 | 0 | 0 | 0 | 0 | 0 | 0 | 0 | 0 | 0 |
| Crocidura attenuata | 1 | 1 | 1 | 1 | 0 | 1 | 1 | 1 | 1 | 0 | 0 | 0 | 0 |
| Dremomys pernyi | 1 | 1 | 1 | 0 | 0 | 0 | 1 | 1 | 0 | 0 | 0 | 0 | 1 |
| Dremomys pyrrhomerus | 1 | 1 | 1 | 1 | 0 | 0 | 0 | 0 | 0 | 0 | 0 | 0 | 0 |
| Dremomys rufigenis | 0 | 0 | 0 | 0 | 0 | 0 | 0 | 0 | 0 | 0 | 0 | 0 | 0 |
| Eothenomys miletus | 0 | 1 | 1 | 0 | 0 | 0 | 1 | 0 | 0 | 0 | 0 | 0 | 0 |
| Eothenomys melanogaster | 1 | 1 | 1 | 1 | 0 | 1 | 1 | 0 | 0 | 0 | 0 | 1 | 1 |
| Leopoldamys edwardsi | 1 | 1 | 1 | 0 | 1 | 1 | 1 | 1 | 0 | 0 | 0 | 1 | 1 |
| Micromys minutus | 1 | 0 | 0 | 0 | 0 | 0 | 0 | 1 | 0 | 0 | 0 | 1 | 0 |
| Mogera insularis | 1 | 1 | 0 | 0 | 0 | 0 | 0 | 1 | 1 | 0 | 0 | 1 | 0 |
| Mus pahari | 0 | 0 | 0 | 0 | 0 | 0 | 0 | 0 | 0 | 0 | 1 | 0 | 0 |
| Myospalax rothschildi | 0 | 0 | 0 | 0 | 0 | 0 | 0 | 0 | 0 | 0 | 0 | 0 | 0 |
| Niviventer andersoni | 0 | 1 | 0 | 0 | 0 | 0 | 1 | 0 | 0 | 0 | 0 | 0 | 0 |
| Niviventer confucianus | 0 | 1 | 1 | 0 | 1 | 1 | 1 | 0 | 1 | 0 | 0 | 1 | 1 |
| Niviventer fulvescens | 1 | 1 | 1 | 1 | 1 | 1 | 1 | 1 | 1 | 1 | 1 | 1 | 1 |
| Rattus losea | 0 | 0 | 0 | 0 | 1 | 0 | 0 | 0 | 0 | 0 | 0 | 0 | 0 |
| Rattus nitidus | 1 | 1 | 1 | 0 | 0 | 1 | 1 | 0 | 1 | 0 | 0 | 0 | 1 |
| Rattus norvegicus | 1 | 1 | 1 | 1 | 1 | 1 | 1 | 0 | 0 | 1 | 1 | 1 | 1 |
| Rattus pyctoris | 0 | 0 | 0 | 0 | 0 | 0 | 0 | 0 | 0 | 0 | 0 | 0 | 1 |
| Rattus tanezumi | 1 | 1 | 1 | 1 | 0 | 1 | 1 | 0 | 1 | 0 | 0 | 1 | 1 |
| Scapanulus oweni | 0 | 0 | 0 | 0 | 0 | 0 | 0 | 0 | 0 | 0 | 0 | 0 | 0 |
| Scaptonyx fusicaudus | 0 | 0 | 0 | 0 | 0 | 0 | 0 | 0 | 0 | 0 | 0 | 0 | 0 |
| Sciurotamias davidianus | 0 | 0 | 0 | 0 | 0 | 0 | 0 | 1 | 1 | 1 | 1 | 0 | 0 |
| Sorex bedfordiae | 0 | 0 | 0 | 0 | 0 | 0 | 0 | 0 | 0 | 0 | 0 | 0 | 0 |
| Sorex cylindricauda | 0 | 0 | 0 | 0 | 0 | 0 | 0 | 0 | 0 | 0 | 0 | 0 | 0 |
| Suncus murinus | 1 | 1 | 1 | 0 | 0 | 0 | 0 | 0 | 0 | 0 | 0 | 0 | 0 |
| Euroscaptor longirostris | 0 | 0 | 0 | 1 | 0 | 0 | 0 | 0 | 0 | 0 | 0 | 0 | 0 |
| Tamiops maritimus | 0 | 0 | 0 | 0 | 0 | 0 | 0 | 0 | 0 | 0 | 0 | 0 | 0 |
| Tamiops swinhoei | 1 | 1 | 1 | 0 | 0 | 0 | 1 | 0 | 1 | 0 | 0 | 0 | 1 |
| Typhlomys cinereus | 0 | 1 | 1 | 0 | 0 | 1 | 1 | 1 | 0 | 0 | 0 | 0 | 1 |
| Uropsilus soricipes | 0 | 0 | 1 | 0 | 0 | 0 | 0 | 0 | 0 | 0 | 0 | 0 | 0 |

| site | Mt. Fanjing | | | | Mt. Jinfo | | | | Mt. Qizimei | | | Mt. Dashahe | |
| --- | --- | --- | --- | --- | --- | --- | --- | --- | --- | --- | --- | --- | --- |
|  | 1000m | 1600m | 2000m | 2250m | 700m | 1150m | 1750m | 2250m | 1000m | 1450m | 1850m | 1050m | 1650m |
| Anourosorex squamipes | 1 | 1 | 1 | 0 | 0 | 1 | 1 | 1 | 0 | 1 | 1 | 1 | 1 |
| Apodemus agrarius | 1 | 0 | 0 | 0 | 1 | 1 | 0 | 0 | 1 | 0 | 0 | 1 | 0 |
| Apodemus chevrieri | 0 | 1 | 1 | 0 | 0 | 1 | 1 | 0 | 0 | 0 | 1 | 0 | 0 |
| Apodemus draco | 0 | 1 | 1 | 0 | 0 | 1 | 1 | 0 | 0 | 1 | 1 | 1 | 1 |
| Berylmys bowersi | 1 | 1 | 0 | 0 | 1 | 1 | 1 | 0 | 0 | 0 | 0 | 0 | 0 |
| Blarinella griselda | 0 | 0 | 0 | 0 | 0 | 0 | 0 | 0 | 0 | 0 | 0 | 0 | 0 |
| Blarinella quadraticauda | 1 | 1 | 1 | 0 | 0 | 1 | 1 | 0 | 0 | 1 | 1 | 1 | 1 |
| Callosciurus erythraeus | 0 | 0 | 0 | 0 | 0 | 0 | 1 | 0 | 1 | 1 | 1 | 1 | 1 |
| Chodsigoa parca | 1 | 1 | 1 | 0 | 0 | 0 | 0 | 0 | 0 | 0 | 0 | 0 | 0 |
| Crocidura attenuata | 1 | 1 | 0 | 0 | 0 | 1 | 1 | 1 | 1 | 0 | 0 | 0 | 0 |
| Dremomys pernyi | 0 | 1 | 0 | 0 | 0 | 0 | 1 | 0 | 0 | 0 | 1 | 0 | 1 |
| Dremomys pyrrhomerus | 0 | 0 | 0 | 0 | 0 | 0 | 0 | 0 | 0 | 0 | 0 | 0 | 0 |
| Dremomys rufigenis | 0 | 0 | 0 | 0 | 0 | 0 | 1 | 0 | 0 | 1 | 0 | 1 | 0 |
| Eothenomys miletus | 0 | 1 | 1 | 1 | 0 | 0 | 0 | 1 | 0 | 1 | 1 | 1 | 0 |
| Eothenomys melanogaster | 1 | 1 | 1 | 1 | 0 | 0 | 0 | 0 | 0 | 1 | 1 | 0 | 0 |
| Leopoldamys edwardsi | 1 | 1 | 0 | 0 | 1 | 1 | 1 | 0 | 1 | 0 | 0 | 1 | 1 |
| Micromys minutus | 1 | 1 | 0 | 0 | 0 | 1 | 0 | 0 | 1 | 0 | 0 | 1 | 0 |
| Mogera insularis | 1 | 0 | 0 | 0 | 0 | 0 | 0 | 0 | 0 | 0 | 0 | 0 | 0 |
| Mus pahari | 1 | 0 | 0 | 0 | 0 | 1 | 1 | 0 | 0 | 0 | 0 | 0 | 0 |
| Myospalax rothschildi | 0 | 0 | 0 | 0 | 0 | 0 | 1 | 1 | 0 | 0 | 0 | 0 | 0 |
| Niviventer andersoni | 0 | 0 | 0 | 0 | 0 | 0 | 0 | 1 | 0 | 0 | 0 | 0 | 1 |
| Niviventer confucianus | 1 | 1 | 1 | 0 | 1 | 1 | 1 | 0 | 1 | 1 | 1 | 1 | 1 |
| Niviventer fulvescens | 1 | 1 | 1 | 1 | 1 | 1 | 1 | 1 | 1 | 1 | 0 | 0 | 0 |
| Rattus losea | 0 | 0 | 0 | 0 | 1 | 1 | 1 | 0 | 0 | 0 | 0 | 0 | 0 |
| Rattus nitidus | 1 | 1 | 1 | 1 | 1 | 1 | 0 | 0 | 1 | 1 | 1 | 0 | 0 |
| Rattus norvegicus | 1 | 0 | 0 | 0 | 1 | 1 | 0 | 0 | 1 | 0 | 0 | 1 | 1 |
| Rattus pyctoris | 1 | 0 | 0 | 0 | 1 | 1 | 1 | 0 | 0 | 0 | 0 | 0 | 0 |
| Rattus tanezumi | 1 | 1 | 0 | 0 | 0 | 1 | 0 | 0 | 1 | 1 | 1 | 1 | 1 |
| Scapanulus oweni | 0 | 0 | 0 | 0 | 0 | 0 | 0 | 0 | 0 | 0 | 0 | 0 | 0 |
| Scaptonyx fusicaudus | 0 | 1 | 1 | 0 | 1 | 1 | 1 | 0 | 0 | 0 | 0 | 0 | 1 |
| Sciurotamias davidianus | 0 | 1 | 0 | 0 | 0 | 0 | 1 | 1 | 1 | 1 | 1 | 1 | 1 |
| Sorex bedfordiae | 0 | 0 | 1 | 0 | 0 | 0 | 0 | 0 | 0 | 0 | 0 | 0 | 0 |
| Sorex cylindricauda | 0 | 0 | 0 | 0 | 0 | 0 | 0 | 0 | 0 | 0 | 0 | 0 | 0 |
| Suncus murinus | 1 | 1 | 0 | 0 | 0 | 0 | 0 | 0 | 0 | 0 | 0 | 0 | 0 |
| Euroscaptor longirostris | 0 | 0 | 0 | 0 | 1 | 1 | 1 | 0 | 1 | 1 | 0 | 0 | 0 |
| Tamiops maritimus | 0 | 1 | 0 | 0 | 0 | 0 | 0 | 0 | 0 | 0 | 0 | 0 | 0 |
| Tamiops swinhoei | 1 | 1 | 0 | 0 | 0 | 1 | 0 | 0 | 0 | 0 | 1 | 0 | 0 |
| Typhlomys cinereus | 0 | 1 | 1 | 0 | 0 | 0 | 1 | 0 | 1 | 0 | 0 | 0 | 0 |
| Uropsilus soricipes | 1 | 1 | 1 | 0 | 0 | 1 | 1 | 0 | 0 | 0 | 0 | 0 | 0 |

| site | Mt. Xingdou (Liu, 2013) | | | Mt. Mulinzi (Ge, 2013) | |
| --- | --- | --- | --- | --- | --- |
|  | 800m | 1250m | 1600m | 1000m | 1500m |
| Anourosorex squamipes | 0 | 1 | 1 | 0 | 1 |
| Apodemus agrarius | 1 | 0 | 0 | 0 | 0 |
| Apodemus chevrieri | 0 | 0 | 1 | 0 | 1 |
| Apodemus draco | 0 | 0 | 1 | 0 | 1 |
| Berylmys bowersi | 0 | 0 | 0 | 0 | 0 |
| Blarinella griselda | 0 | 0 | 0 | 0 | 0 |
| Blarinella quadraticauda | 0 | 1 | 1 | 1 | 1 |
| Callosciurus erythraeus | 0 | 1 | 1 | 1 | 1 |
| Chodsigoa parca | 0 | 0 | 0 | 0 | 1 |
| Crocidura attenuata | 0 | 1 | 1 | 1 | 1 |
| Dremomys pernyi | 0 | 0 | 1 | 0 | 1 |
| Dremomys pyrrhomerus | 0 | 0 | 0 | 0 | 0 |
| Dremomys rufigenis | 1 | 1 | 0 | 1 | 0 |
| Eothenomys miletus | 0 | 1 | 1 | 0 | 1 |
| Eothenomys melanogaster | 0 | 1 | 1 | 1 | 1 |
| Leopoldamys edwardsi | 1 | 1 | 1 | 1 | 1 |
| Micromys minutus | 0 | 0 | 0 | 1 | 0 |
| Mogera insularis | 0 | 0 | 0 | 0 | 0 |
| Mus pahari | 0 | 0 | 0 | 0 | 0 |
| Myospalax rothschildi | 0 | 1 | 1 | 0 | 1 |
| Niviventer andersoni | 0 | 0 | 0 | 0 | 0 |
| Niviventer confucianus | 1 | 1 | 1 | 1 | 1 |
| Niviventer fulvescens | 1 | 1 | 1 | 1 | 1 |
| Rattus losea | 0 | 0 | 0 | 1 | 0 |
| Rattus nitidus | 1 | 1 | 1 | 1 | 1 |
| Rattus norvegicus | 1 | 1 | 1 | 1 | 1 |
| Rattus pyctoris | 0 | 0 | 0 | 0 | 0 |
| Rattus tanezumi | 0 | 1 | 1 | 1 | 1 |
| Scapanulus oweni | 0 | 0 | 0 | 0 | 0 |
| Scaptonyx fusicaudus | 0 | 0 | 0 | 0 | 0 |
| Sciurotamias davidianus | 0 | 1 | 1 | 0 | 0 |
| Sorex bedfordiae | 0 | 0 | 0 | 0 | 0 |
| Sorex cylindricauda | 0 | 0 | 0 | 1 | 1 |
| Suncus murinus | 0 | 0 | 0 | 0 | 0 |
| Euroscaptor longirostris | 0 | 0 | 1 | 0 | 0 |
| Tamiops maritimus | 0 | 0 | 0 | 0 | 0 |
| Tamiops swinhoei | 0 | 0 | 1 | 0 | 1 |
| Typhlomys cinereus | 0 | 0 | 0 | 0 | 0 |
| Uropsilus soricipes | 0 | 0 | 0 | 0 | 0 |

Ge, J. W., Hu, H. X., Li, B. (2009) Scientific survey and study on forest biodiversity in Mulinzi Nature Reserve of Hubei, central China.

Liu, S. X., Qu, J. P. (2013). Scientific survey of Xingdoushan Nature Reserve, Hubei.

Table S2 Environment variables in every elevation gradient

| elevation | 600 | 800 | 1000 | 1200 | 1400 | 1600 | 1800 | 2000 | 2200 |
| --- | --- | --- | --- | --- | --- | --- | --- | --- | --- |
| AMT | 15.95851 | 15.06593 | 14.17569 | 13.19812 | 12.0887 | 11.00288 | 10.21273 | 9.190833 | 8.529167 |
| AP | 1317.319 | 1296.389 | 1301.631 | 1322.365 | 1344.853 | 1377.19 | 1370.63 | 1416 | 1399.75 |
| NPP | 2.56E+11 | 2.57E+11 | 2.56E+11 | 2.58E+11 | 2.64E+11 | 2.66E+11 | 2.61E+11 | 2.85E+11 | 2.53E+11 |
| HII | 19.32861 | 18.69068 | 17.87373 | 17.14349 | 16.28772 | 15.48 | 14.81481 | 18 | 16 |
| NDVI | 25.46942 | 25.38361 | 25.2556 | 25.60614 | 26.16637 | 27.76083 | 28.59105 | 33.5 | 29.6875 |

Table S3 Accession number of every species from GenBank.

| species | accession number |
| --- | --- |
| *Anourosorex squamipes* | KT032946 |
| *Apodemus agrarius* | AB096817 |
| *Apodemus chevrieri* | AB096819 |
| *Apodemus draco* | MK329550 |
| *Berylmys bowersi* | JX573336 |
| *Blarinella griselda* | JF719723 |
| *Blarinella quadraticauda* | JF719721 |
| *Callosciurus erythraeus* | HQ698359 |
| *Chodsigoa parca* | KX765508 |
| *Crocidura attenuata* | AB175083 |
| *Dremomys pernyi* | HQ698362 |
| *Dremomys pyrrhomerus* | EF539342 |
| *Dremomys rufigenis* | EF539341 |
| *Eothenomys miletus* | HM165379 |
| *Eothenomys melanogaster* | KX113462 |
| *Leopoldamys edwardsi* | KY068766 |
| *Micromys minutus* | AB201996 |
| *Mogera insularis* | AB037606 |
| *Mus pahari* | AB096839 |
| *Myospalax rothschildi* | AF326268 |
| *Niviventer andersoni* | KP754658 |
| *Niviventer confucianus* | KP754665 |
| *Niviventer fulvescens* | KY068838 |
| *Rattus losea* | HM031721 |
| *Rattus nitidus* | MG748342 |
| *Rattus norvegicus* | LC147012 |
| *Rattus pyctoris* | MG748340 |
| *Rattus tanezumi* | KY002826 |
| *Scapanulus oweni* | KC192652 |
| *Scaptonyx fusicaudus* | KX754478 |
| *Sciurotamias davidianus* | KC005710 |
| *Sorex bedfordiae* | KJ547342 |
| *Sorex cylindricauda* | KJ547381 |
| *Suncus murinus* | JF784171 |
| *Euroscaptor longirostris* | HG737871 |
| *Tamiops maritimus* | HQ698387 |
| *Tamiops swinhoei* | EF539334 |
| *Typhlomys cinereus* | KX397283 |
| *Uropsilus soricipes* | KF778194 |

Table S4 Functional traits of each species

| *Species* | Body Mass | Body Length | Tail Length | Hind Foot Length | Ear Length | specimen ID | Foraging stratum | Activity time | Diet | Habitat type |
| --- | --- | --- | --- | --- | --- | --- | --- | --- | --- | --- |
| *Anourosorex squamipes* | 20 | 92 | 13.5 | 13.5 | 0 | QZM16043 | ground | nocturnal | invertebrate | terrestrial |
| *Apodemus agrarius* | 23.3 | 96.5 | 93.5 | 20.5 | 13.5 | MM140155 | ground | diurnal | herbivorous | terrestrial |
| *Apodemus chevrieri* | 32.9 | 99 | 94 | 23.5 | 15 | GZ15196 | ground | diurnal | herbivorous | terrestrial |
| *Apodemus draco* | 22.25 | 96.5 | 91 | 21.5 | 16 | GZ15201 | ground | multi-time | herbivorous | terrestrial |
| *Berylmys bowersi* | 300 | 260.5 | 270.5 | 54.5 | 34 | GZ18116 | ground | nocturnal | herbivorous | terrestrial |
| *Blarinella griselda* | 12.5 | 65.5 | 36.5 | 11 | 6 | HN2017152 | ground | nocturnal | invertebrate | terrestrial |
| *Blarinella quadraticauda* | 12.5 | 73 | 50 | 14.5 | 21 | QZM16052 | ground | nocturnal | invertebrate | terrestrial |
| *Callosciurus erythraeus* | 280 | 207.5 | 206.5 | 48 | 20.5 | SCH05055 | arboreal | diurnal | herbivorous | arboreal |
| *Chodsigoa parca* | 5.49 | 76 | 91 | 17.5 | 9 | BZC452 | ground | nocturnal | invertebrate | terrestrial |
| *Crocidura attenuata* | 14.27 | 74.5 | 50.5 | 13.5 | 10 | QZM16024 | ground | nocturnal | invertebrate | terrestrial |
| *Dremomys pernyi* | 198.63 | 200 | 168 | 48.5 | 23.5 | MM140119 | scansorial | diurnal | herbivorous | terrestrial |
| *Dremomys pyrrhomerus* | 167.73 | 202.5 | 151 | 52.5 | 23 | W306 | scansorial | diurnal | herbivorous | terrestrial |
| *Dremomys rufigenis* | 200 | 199 | 155 | 49 | 24 | 6398 | scansorial | diurnal | herbivorous | arboreal |
| *Eothenomys miletus* | 50.7 | 115 | 45 | 19.5 | 13.5 | GZ15005 | ground | multi-time | herbivorous | terrestrial |
| *Eothenomys melanogaster* | 27 | 97.5 | 31.5 | 16 | 11 | BD16230 | ground | multi-time | herbivorous | terrestrial |
| *Leopoldamys edwardsi* | 300 | 250 | 289.5 | 50 | 30 | BD16252 | ground | nocturnal | invertebrate | terrestrial |
| *Micromys minutus* | 6 | 61.5 | 66.5 | 15 | 10 | GZ15146 | ground | multi-time | herbivorous | terrestrial |
| *Mogera insularis* | 83.03 | 112 | 8.5 | 11.5 | 0 | CQ18197 | ground | multi-time | invertebrate | terrestrial |
| *Mus pahari* | 25.73 | 95.5 | 89 | 22 | 16 | GZ18180 | ground | nocturnal | herbivorous | terrestrial |
| *Myospalax rothschildi* | 302 | 160.5 | 33 | 27 | 6.5 | TB07130 | ground | nocturnal | herbivorous | terrestrial |
| *Niviventer andersoni* | 147 | 174 | 231.5 | 35.5 | 25 | HN2017257 | scansorial | nocturnal | herbivorous | terrestrial |
| *Niviventer confucianus* | 65 | 144.5 | 204.5 | 31.5 | 23 | HN2017283 | scansorial | nocturnal | herbivorous | terrestrial |
| *Niviventer fulvescens* | 82.55 | 151.5 | 190.5 | 32 | 23 | HN2017288 | scansorial | nocturnal | herbivorous | terrestrial |
| *Rattus losea* | 77 | 152.5 | 151.5 | 28 | 19.5 | BQ004 | ground | nocturnal | herbivorous | terrestrial |
| *Rattus nitidus* | 133.5 | 164 | 170.5 | 34 | 21 | MB10010 | ground | nocturnal | herbivorous | terrestrial |
| *Rattus norvegicus* | 338.33 | 232.5 | 220 | 44 | 22.5 | QZM16161 | ground | nocturnal | herbivorous | terrestrial |
| *Rattus pyctoris* | 149.96 | 152.5 | 156.5 | 33 | 22.5 | GZ18233 | ground | nocturnal | herbivorous | terrestrial |
| *Rattus tanezumi* | 134 | 160 | 175 | 30.5 | 20 | GZ18319 | ground | nocturnal | herbivorous | terrestrial |
| *Scapanulus oweni* | 36.64 | 122 | 39 | 17 | 0 | PB10106 | ground | multi-time | invertebrate | terrestrial |
| *Scaptonyx fusicaudus* | 19.41 | 81 | 35.5 | 17 | 0 | HZ2017013 | ground | multi-time | invertebrate | terrestrial |
| *Sciurotamias davidianus* | 433.51 | 220 | 162.5 | 52 | 24 | GZ15113 | ground | diurnal | herbivorous | terrestrial |
| *Sorex bedfordiae* | 6.6 | 61 | 57 | 13 | 4.5 | WL16110 | ground | multi-time | invertebrate | terrestrial |
| *Sorex cylindricauda* | 6.05 | 72 | 58.5 | 15.5 | 6 | GZ18325 | ground | multi-time | invertebrate | terrestrial |
| *Suncus murinus* | 60.71 | 146.5 | 72.5 | 20.5 | 12 | GZ18304 | ground | nocturnal | invertebrate | terrestrial |
| *Euroscaptor longirostris* | 59.57 | 117.5 | 18 | 18.5 | 0 | WL15004 | ground | multi-time | invertebrate | terrestrial |
| *Tamiops maritimus* | 70.33 | 119.5 | 97.5 | 27.5 | 13 | 14917 | arboreal | diurnal | herbivorous | arboreal |
| *Tamiops swinhoei* | 85 | 152 | 91.5 | 31.5 | 12.5 | GZ18097 | arboreal | diurnal | herbivorous | arboreal |
| *Typhlomys cinereus* | 31.2 | 78.5 | 119 | 21 | 15.5 | BD16200 | arboreal | nocturnal | herbivorous | terrestrial |
| *Uropsilus soricipes* | 16 | 73 | 59.5 | 15.5 | 10.5 | HN2017013 | ground | multi-time | invertebrate | terrestrial |

Note: The data of ear length, hind foot length, tail length, body length and body mass are measured from specimen in Institution of Zoology, Chinese Academy of Sciences, Beijing, China (IOZCAS). The diet, habitat, foraging stratum and daily activity are extracted from Wilman et al., (2014).

Wilman, H., Belmaker, J., Simpson, J., de la Rosa, C., Riv adeneira, M. M., & Jetz, W. (2014). EltonTraits 1.0: Species-level foraging attributes of the world's birds and mammals. *Ecology*, 95(7), 2027-2027. https://doi.org/10.1890/13-1917.1.

Table S5 Phylogenetic conservatism tests for 39 small mammal functional traits of Wuling Mountains.

| Traits | K | P-value | Traits | D | P (D > 0) | P (D < 1) | Traits | r.Mantel | p.null | p.BM |
| --- | --- | --- | --- | --- | --- | --- | --- | --- | --- | --- |
| BodyMass | 0.761126 | 0.001 | Diet | 1.16465 | 0.998 | 0 | Foraging stratum | 0.1889 | 0.0034 | 0.1235 |
| BodyLength | 0.822504 | 0.001 | Habitat | 1.32014 | 0.975 | 0 | Activity time | 0.2833 | 0.0002 | 0.0939 |
| TailLength | 1.571967 | 0.001 |  |  |  |  |  |  |  |  |
| HindFootLength | 1.011079 | 0.001 |  |  |  |  |  |  |  |  |
| EarLength | 1.265885 | 0.001 |  |  |  |  |  |  |  |  |

Table S6 Three diversity facets in overall species (all), broad range species (br), narrow range species (nr), rodentia (rod) and eulipotyphla (eu).

| elevation | 600 | 800 | 1000 | 1200 | 1400 | 1600 | 1800 | 2000 | 2200 |
| --- | --- | --- | --- | --- | --- | --- | --- | --- | --- |
| TDall | 7 | 13 | 30 | 32 | 36 | 31 | 24 | 15 | 9 |
| PDall | 0.398782 | 0.526155 | 0.660596 | 0.647393 | 0.650972 | 0.647522 | 0.680178 | 0.67017 | 0.654226 |
| FDall | 0.245968 | 0.276288 | 0.30785 | 0.298741 | 0.310976 | 0.295709 | 0.316282 | 0.274543 | 0.266554 |
| TDbr | 7 | 11 | 20 | 20 | 22 | 22 | 18 | 13 | 8 |
| PDbr | 0.401093 | 0.506049 | 0.673595 | 0.673595 | 0.663391 | 0.663391 | 0.696188 | 0.680692 | 0.64925 |
| FDbr | 0.256482 | 0.254965 | 0.318326 | 0.318326 | 0.314222 | 0.314222 | 0.334263 | 0.292473 | 0.284826 |
| TDnr | 0 | 2 | 10 | 12 | 14 | 9 | 6 | 2 | 1 |
| PDnr | NA | 0.822347 | 0.708765 | 0.669944 | 0.689242 | 0.668014 | 0.662474 | 0.691397 | NA |
| FDnr | NA | 0.52393 | 0.34149 | 0.310579 | 0.342403 | 0.302104 | 0.307565 | 0.342118 | NA |
| TDrod | 6 | 10 | 19 | 21 | 25 | 22 | 17 | 8 | 7 |
| PDrod | 0.2692 | 0.339237 | 0.538006 | 0.519355 | 0.543148 | 0.537591 | 0.58881 | 0.538276 | 0.570409 |
| FDrod | 0.234087 | 0.253293 | 0.318392 | 0.307377 | 0.322868 | 0.309369 | 0.327122 | 0.27691 | 0.279724 |
| TDeu | 1 | 3 | 11 | 11 | 11 | 9 | 7 | 7 | 2 |
| PDeu | NA | 0.554341 | 0.671973 | 0.671577 | 0.671577 | 0.693718 | 0.730276 | 0.684482 | 0.763325 |
| FDeu | NA | 0.095238 | 0.077922 | 0.077922 | 0.077922 | 0.071429 | 0.068027 | 0.081633 | 0 |

Table S7 index of SEM

|  | overall | broad_range | narrow_range | rodentia | eulipotyphla |
| --- | --- | --- | --- | --- | --- |
| CFI | 1 | 1 | 1 | 1 | 1 |
| RMSEA | 0 | 0 | 0 | 0 | 0 |
| SRMR | 0 | 0 | 0 | 0 | 0 |
| AIC | -1.128 | 2.129 | 50.431 | -4.033 | 85.708 |

Fig. S1 Elevation pattern of environmental factors in the Wuling Mountain area.

Fig. S2 Phylogenetic tree based on CytB gene including the small mammal species found in the Wuling Mountain area.
